# Gender Differences and Unfairness Processing during Economic and Moral Decision-Making: A fNIRS Study

**DOI:** 10.3390/brainsci10090647

**Published:** 2020-09-17

**Authors:** Maria Elide Vanutelli, Francesca Meroni, Giulia Fronda, Michela Balconi, Claudio Lucchiari

**Affiliations:** 1 Department of Philosophy, Università degli Studi di Milano, 20122 Milan, Italy; francesca.meroni92@gmail.com (F.M.); claudio.lucchiari@unimi.it (C.L.); 2 Research Unit in Social and Affective Neuroscience, Catholic University of Milan, 20122 Milan, Italy; giulia.fronda@unicatt.it (G.F.); michela.balconi@unicatt.it (M.B.); 3 Department of Psychology, Catholic University of Milan, 20122 Milan, Italy

**Keywords:** decision-making, fNIRS, unfairness, ultimatum game, prefrontal cortex

## Abstract

Decisional conflicts have been investigated with social decision-making tasks, which represent good models to elicit social and emotional dynamics, including fairness perception. To explore these issues, we created two modified versions of the UG framed within an economic vs. a moral context that included two kinds of unfair offers: advantageous (upside, U) or disadvantageous (downside, D) from the responder’s perspective, and vice-versa for the proponent. The hemodynamic activity of 36 participants, 20 females and 16 males, was continuously recorded with fNIRS to investigate the presence of general or specific circuits between the different experimental conditions. Results showed that disadvantageous offers (D) are associated with an increased widespread cortical activation. Furthermore, we found that advantageous moral choices at the expense of others (U) were related to the activation of the right prefrontal cortex. Finally, we found gender-related differences in brain activations in the different frameworks. In particular, the DLPFC was recruited by females during the economic task, and by males during the moral frame. In conclusion, the present study confirmed and expanded previous data about the role of the prefrontal cortices in decision-making, suggesting the need for further studies to understand better the different prefrontal networks serving moral and economic decisions also considering gender-related differences.

## 1. Introduction

Decision-making has always been a topic of interest for psychologists as well as scholars such as economists, mathematicians, and all those researchers interested in understanding the human ability to choose the best option in different contexts. In the beginning, the typical paradigm to study such mechanisms consisted of an individual setting where people were required to make decisions solely according to their own criteria. Typically, experimental tasks have been used to elicit decisions about monetary choices or math-based exercises. However, as already suggested by Rilling and Sanfey [1], we do not make decisions purely by ourselves, since we are continuously immersed within a network of social relationship where choices and decisions are shared and negotiated. In these cases, decisions are situated in a given social frame and can be considered the result of both selves- and other-regarding preferences [2].

Previous research already underlined that, when dealing with decisions, people do not behave as pure rational decision-makers who pursue well-defined and convenient options according to certain axioms [3], as suggested by normative theories [4]. Actually, emotional components often influence decisions, and this is particularly true in the case of situated decision-making, which can involve psychological and moral conflicts. In particular, it is crucial to reach a balance between one’s own interests and those of others, between immediate or delayed rewards, and between emotion and reason [1,5,6].

Decisional conflicts have been investigated by using social decision-making tasks [1,7] that can trigger social and emotional cognition, such as considering one’s own and others’ perspectives [8]. Social interactions often require both emotional and “rational” cognitive motivations [7], which may be investigated using social decision-making tasks such as the Ultimatum Game (UG). The UG has been consistently used to explore social decision-making and to analyze the interplay between utilitarianism and the response to fairness, including both economic and moral issues. In detail, the UG involves two players who must share a given amount of money. One player acts as a proposer and the other one as a responder. The proposer is directly given an amount of money and must choose how to split it with the responder. If the responder accepts, both players receive the amount proposed; if he/she refuses, neither player gets any money. It is easy to conclude that a rational responder motivated purely by self-interest should accept any amount offered by the proposer, as this will represent a gain that is anyhow better than zero. Subsequently, a rational proposer could offer the smallest non-zero amount allowed by the game rules [9]. However, evidence shows that people do not follow these rules. When the offer is 20% or below the initial amount, it is rejected about half of the times [10]. Once again, the pattern of choices in the UG reveals that people are not driven only by self-interest since others’ actions are also evaluated based on social fairness. Indeed, when the same offers are made in a control condition, typically where it is clear that the proposal has been computer-generated, rejection rates fall close to zero [11,12].

As proposed by Gaertig and colleagues [13], the decision to reject unfair offers can be explained by several factors, such as inequity aversion [14] and the perception of unfairness, which is accompanied by negative emotions. When presented with an unfair offer, responders often feel wounded pride. Subsequently, they are motivated to punish their game partner even if it is disadvantageous for monetary gains since it is gratifying from an emotional point of view. This mechanism of punishing unfairness can be considered as an adaptive mechanism within a psycho-social framework of fair cooperation. However, even if different psycho-social scenarios modulate such interpersonal dynamics, it is still not clear how the experimental manipulations can specifically affect the perception of fairness when other variables outside the economic framework are introduced in the experimental setting.

Also, even if previous literature mainly focused on unfair offers from the perspective of the receiver (see, for example References [11,15]), it would also be essential to include advantageous unfairness in the model. A few previous studies on adults [16] and children [17] suggested that we tend to prevent inequity both when we receive less and when we receive more. Nonetheless, since this last behavior appears just after 8 years of age, it has been proposed that disadvantageous and advantageous inequity aversion may be subserved by different mechanisms [17]. However, this issue has not yet received the attention it deserves.

From a neuro-cognitive point of view, studies suggested that the prefrontal cortex (PFC) may act as a monitor for either conflict in information processing or action-outcome values [18,19], and different portions of this area are thought to subserve specific functions during decision-making. In particular, the dorsolateral prefrontal cortex (DLPFC) has been hypothesized to have at least three distinctive roles [20]: (A) to provide cognitive control to moderate the arousing social-emotional responses elicited by the social problems [21]; (B) to promote abstract reasoning (e.g., cost-benefit comparison) [21,22]; (C) to favor self-centered and other-aversive emotions [20,23]. Indeed, the DLPFC was found pivotal for adaptive decision making, since it facilitates cautious over riskier choices [24]. For example, previous TMS studies demonstrated that the suppression of this area increases risk-taking behaviors [24,25]. Another area of interest is the medial prefrontal cortex (mPFC), whose activity is related to the magnitude of possible gains and, thus, reward [26]. On the other side, the lateral part of the orbitofrontal cortex (lOFC) is involved in the encoding of outcome-value associations [27]. More specifically, the magnitude of the BOLD response over this area when taking a decision may reflect the risk value assigned based on previous experience. Interestingly, it was demonstrated that the larger the difference in activation between risky and safe choices, the better the final performance [28]. However, differently from the mPFC, this area is more involved in punishment processing [29,30]. In fact, a previous study by O’Doherty and colleagues showed a clear dissociation within the OFC, with the medial parts correlating more with monetary gain, while the lateral parts with how much money is lost [29].

Another critical aspect of studying decision-making is related to recognizing the individual factors that drive our choices, such as gender. Previous research has widely discussed the presence of gender-related behavioral patterns in decision-making. Males and females are thought to follow different strategies. For example, females were found more empathetic and more inclined to follow deontological principles [30,31,32]. In contrast, males are supposed to be more rational and more prone to deal pragmatically with trade-offs despite the risk of harming others [33]. Moreover, other differences were found about risk tolerance [34,35,36,37]. For example, one of the most frequent results is that females are less risk-seeking and more risk aversive than males [38].

On the other hand, males are thought to take more risky decisions in an attempt to maximize their gains, but eventually, they also pay more for the consequences of their choices [35]. Also, it is well-known that males and females react differently to emotions, with females being more accurate in processing, labeling [39], and recognizing [40] facial expression compared to males. Such specificities have also been related to neuro-anatomical differences in brain networks devolved to affective processing in terms, for example, of wider grey-matter volume in specific parts of the limbic system [41]. However, the variety of the experimental tasks and the manipulated variables often led to inconsistent gender-related results across studies [10,42].

Nonetheless, despite the large number of studies addressing gender-related differences in decision-making, no previous study directly compared economic and moral scenarios in a complex setting, which also included a different kind of unfairness. Those different frames could trigger specific neural networks.

Thus, given the available literature discussed so far, the present study aimed at comparing females’ and males’ responses directly during a decision-making task with different frameworks involving economic and moral contexts. Also, we aimed at exploring the presence of differences in the neural networks involved to process advantageous and disadvantageous unfair offers. To do so, we created two modified versions of the UG where the computer proposed the offers after describing different situations involving splitting money for a job done together with a colleague (economic framework, E) or to support a charitable association in support of a colleague’s family facing health issues (moral, M). We pushed participants to pick an option within unfair conditions, thus engendering conflict between the possibility to gain money, thus tolerating the unfairness, and to refuse the money. We proposed both advantageous and disadvantageous choices about the responder (from here on the upside [U] vs. downside [D], respectively). In the former, the offer was unfair from the side of the proposer, so that the responder had to decide if exploiting the given opportunity even though unfair for the partner. Disadvantageous offers (downside: D), instead, were unfair for the responders. Both cases elicit psycho-social and emotional evaluations, but specific PFC activations might process them. Furthermore, since we used the same schema with two different frames (a moral and an economic one), it was possible to verify if specific decision conflicts are differently processed when different scenarios are presented. Indeed, during the two subtasks, participants’ neural activity was continuously recorded by functional near-infrared spectroscopy (fNIRS).

fNIRS is a functional neuroimaging technology that measures concentration changes of oxygenated (O2Hb) and deoxygenated-hemoglobin (HHb) at the level of cortical microcirculation blood vessels [43]. It allows interesting applications in social neuroscience experiments since, differently from other imaging techniques, it is noninvasive, silent, portable, and does not impose physical constraints [44,45].

In light of previous work discussed above, the starting hypothesis of the present experiment is that making decisions in different frames (economic vs. moral) and responding to various types of offers (upside vs. downside) modulate the PFC network differently. We hypothesized that disadvantageous (downside: D) and advantageous (upside: U) offers would be associated with different activation patterns. Previous literature mainly focused on the differences between fair and unfair offers. However, the novelty of the present study was to compare the neural correlates of unfair offers concerning both upside and downside offers. In detail, we expected that unfair proposals could be associated with increased cognitive conflict and, subsequently, cognitive load that could result in a general prefrontal activation. Indeed, participants know that, if they reject the offer, they lose all the money, and so they need to calculate the pros and cons correctly. On the other hand, we expected that upside offers would be associated with more emotion-related processing, especially for females, since accepting more than the opponent could elicit prosocial reflections and unfairness avoidance [46,47,48].

We further hypothesized differences within the moral frames, since, in this context, the DLPFC is modulated by other cortical and subcortical areas to take into consideration the different cognitive, emotional, and social aspects. In particular, we hypothesized that the lOFC would be particularly active in regulating the DLPFC activity and avoiding fast (impulsive) decisions. Based on previous research [31,46,49], it may be supposed that females will show more altruistic behaviors, while males would be more self-focused. Furthermore, other evidence suggests that males override intuitive moral options in highly emotional and challenging moral dilemmas more often than females do [31]. This might imply that males are rather pragmatic despite the risk of harming others.

On the other hand, females seem to be more empathetic and to care for others at risk. Finally, in agreement with previous studies [46], we might expect females to be more cognitively involved in moral tasks and that this will probably affect their responses. Indeed, some studies reported females to be more affected by the specific features of the experimental conditions [50]. Considering all these differences in moral decision making, we hypothesized that the prefrontal network recruited by our task would be differently modulated due to gender factors. In particular, we expected to find differences in the role of the DLPFC in evaluating moral frames between males and females. Since the DLPFC is particularly implied in cognitively demanding tasks, we hypothesized a relatively higher activation in females than in males. However, the lOFC might also be more active in females than in males due to a more general evaluation of emotional and prosocial considerations. Thus, we expect that the prefrontal network activity will be modulated by the different circuits competing for the final decision.

## 2. Materials and Methods

### 2.1. Participants

Thirty-six subjects, 20 females and 16 males participated in the experiment. Four subjects, 1 woman and 3 males were excluded from the statistical analyses due to the presence of an excessive number of artifacts. Thus, the final sample included 32 participants, 19 females and 13 males of comparable age (range = 22–28; M_w_ = 25.26; SD = 2.28; M_m_ = 25.38; SD = 1.26) and education (range = 15–18; M_w_ = 16.95; SD = 1.18; M_m_ = 17.23; SD = 1.59). Differences in age and years of education between males and females were not statistically significant (t = 0.350, *p* = 0.690). Information about and call for participation in the study were published on the involved Universities’ websites. An email and telephone number were provided to allow people to ask for further information and to book an individual meeting. During this first meeting, a researcher explained the study procedures, asked further questions, evaluated the presence of inclusion criteria, and provided the informed consent to be signed before participation.

Inclusion criteria were: not having a history of neurological and psychiatric diseases; agreed to sign the informed consent. Participants were volunteers, and they could withdraw their consent at any time during the study. All participants were right-handed, with normal or corrected-to-normal visual acuity.

The research conduction was approved by the local ethics committee of the Department of Psychology of the Catholic University of the Sacred Heart (a.2017) and has followed the principles and guidelines of the Helsinki Declaration. All the procedures were carried out with an adequate understanding of the subjects, who read and signed the Research Consent Form before participating in this research. No payment was provided.

### 2.2. Procedure

Participants were seated in a dimly lit room facing a computer monitor placed 70 cm from them. Stimuli were presented using E-Prime 2.0 software (Psychology Software Tools, Inc., Sharpsburg, PA, USA) on a personal computer with a 15-inch screen. The experiment was inspired by the Ultimatum Game (UG) and built up using the same structure. Thus, there was a hypothetical bidder who made some different proposals that participants were asked whether to accept or not. According to the original paradigm, subjects were informed that, in case they did not accept, both parts would have lost the amount of the payoff. Participants were asked to read the informed written consent and to sign it. After that, NIRS optodes were positioned as described in Section 2.3. Before the experiment, participants were asked to read the instructions on the screen carefully, which could be completed orally by the researcher if needed. Two different situations composed the task: economic (E) and moral (M). These two situations were presented in random order on the computer monitor and separated by a blank page to allow a brief pause.

Each scenario was built in 3 versions, each presenting a different type of offer that was proposed in a randomized order within each condition. The offers could be neutral (N) or unfair. In the case of a neutral proposal, the money of the E and M situations was equally distributed between the two parts (50%/50%; 49%/51%; 48%/52%). For what concerns the unfair conditions, they could be of two kinds: downside (D) or upside (U). In detail, the D offers led to a disadvantage for the bidder (20%-19%-18% besides the 80%-81%-82% for the colleague or the association), while the U offer led to the opposite outcome (see Table 1).

In the E situation, participants were presented with a scenario in which a colleague asked them for help with an extra-work, which could lead to extra money. They were also informed that, after accepting the job, the contribution from the two parts was substantially equal. Afterword, they were required to accept or refuse some possible offers from the colleague about how to split the earned money, which consisted of 1000€. Nonetheless, they were reminded about the fact that if they had rejected the offer, neither of them would have obtained the money.

In the M condition, to create a neutral offer, participants were required to imagine a situation in which all employees could benefit from a collective special bonus at work in addition to their usual salary because the company is doing well in that period. However, to help a colleague in times of need, they were proposed by their boss to split this bonus with a charity association that supported his son (or her daughter, see later for further details) fighting against leukaemia. Within the M scenario, it is essential to highlight that, differently from U and D, the extra money was not coming from participants’ own work but was extra. In the U and D conditions, however, to reinforce the unbalance, they were told that such money would have been taken from their 1000€ annual bonus, which is given each year. So, in the neutral condition, subjects always obtained a gain, while in the U and D, they suffered a loss.

Thus, the task was composed of 6 blocks, 3 for economic (E), and 3 for moral (M) situations, with neutral (N), downside (D), or upside (U) offers. Each block lasted about 4 min for approximately 40 min overall, including pauses. Each offer was randomly repeated 30 times for a total of 270 repetitions, which also included a linguistic reversal of the two characters (“Marco offers you 80% of the money and he keeps 20% for himself” vs. “Marco offers to keep 20% of the money for himself and propose you 80%”). Participants could make a choice by pressing the “i” and “o” keys to accept or refuse the offer, respectively. Right after a participant took a decision, a “+” appeared in the center of the computer monitor for 5 s to allow the hemodynamic response to reach its peak and go down to baseline values. Sentences were presented on a black background typed in light grey Calibri 20 font.

**Figure 1:**
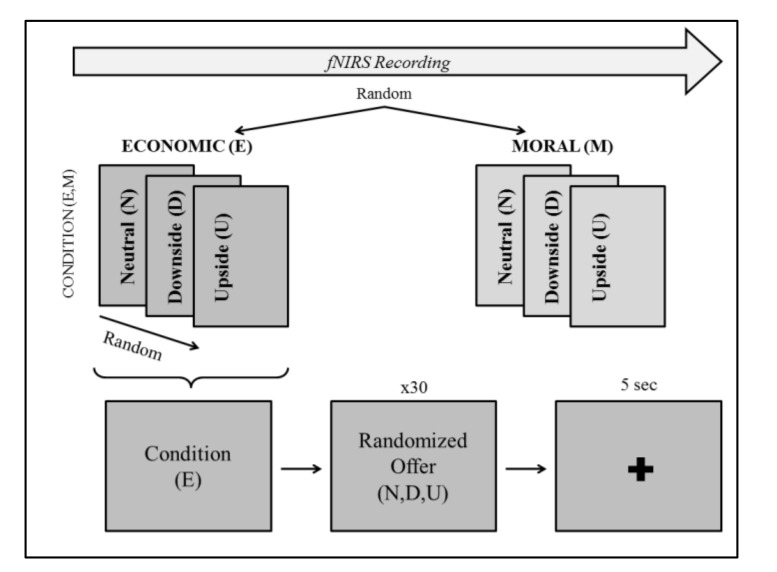
Schematic representation of the structure of the adapted version of the UG used. While fNIRS was recording, Economic (E) and Moral (M) conditions randomly appeared on the computer. Each of them included three different offers (i.e., Neutral (N), Downside (D), Upside (U)) presented in a random order (30 times each, 90 times in total for each condition), which were asked whether to accept or reject those offers. After a decision had been made, a cross appeared in the middle of the screen for 5 s. A short break separated the two conditions.

Moreover, to prevent any possible confounding gender-related effects, two versions of the task were created: one for males (with male names for the bidders) and the other one for females (with female names for the bidders). Before the experiment started, a 5 min-simulation of the paradigm was presented to the participants as a familiarization phase, following the same structure (see Figure 1). Finally, subjects were asked to complete BIS/BAS and BIG-5 questionnaires.

### 2.3. fNIRS Recording and Analyses

**Figure 2:**
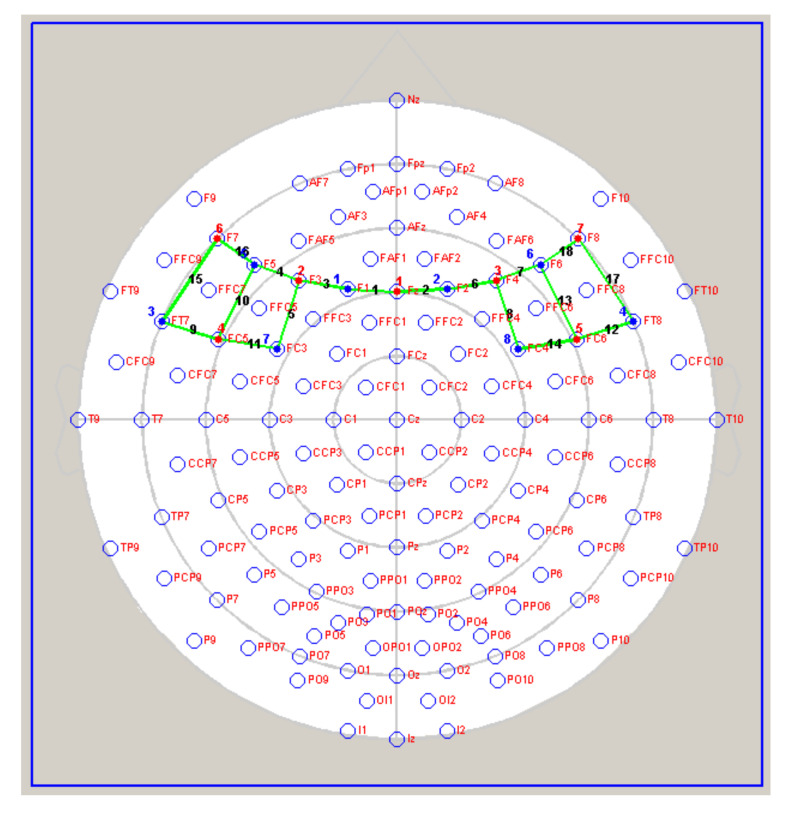
2D representation of the 18-channels array of optodes (7 light sources/emitters and 8 detectors). The emitters are represented by bold red numbers, whereas bold blue numbers represent the detectors. Green lines symbolize the channels created between emitters and optodes. Each channel has its number (i.e., black bold numbers) from 1 to 18.

fNIRS measurements were conducted with NIRScout System (NIRx Medical Technologies, LLC. Los Angeles, CA, USA) using an 18-channels array of optodes (7 light sources/emitters and 8 detectors) covering the dorsolateral prefrontal cortex (DLPFC), the dorsal part of the medial prefrontal cortex (dmPFC) and the lateral orbitofrontal cortex (lOFC). Sources were placed on positions Fz-F3-F3-F4-F7-F8 and FC5-FC6, while detectors were placed on F1-F2-F5-F6, FC3-FC4, and FT7-FT8 (see Figure 2).

The Emitter-detector distance was 30 mm for contiguous optodes, and near-infrared light of two wavelengths (760 and 850 nm) was used. NIRS optodes were attached to the subject’s head using a NIRS cap (international 10/5 system; [51]). With NIRStar acquisition software, changes in the concentration of oxygenated (O2Hb) and deoxygenated hemoglobin (HHb) were recorded continuously throughout the paradigm. Signals obtained from the 18 NIRS channels were measured with a sampling rate of 6.25 Hz, analyzed and transformed with nirsLAB software (v2014.05; NIRx Medical Technologies LLC, 15 Cherry Lane, Glen Head, NY, USA), according to their wavelength and location, resulting in values for the changes in the concentration of oxygenated and deoxygenated hemoglobin for each channel. The raw data of O2Hb and HHb from individual channels were digitally band-pass filtered at 0.01–0.3 Hz. The neutral condition (N) was used as a baseline. Then, the mean concentration of U and D offers was calculated for each condition by creating specific indices as the difference of the means of the baseline (N; m1) and condition (m2) divided by the standard deviation (s) of the baseline: d = (m1 − m2)/s. To interpret the event-related responses to stimuli concerning the baseline, we inverted signs. Finally, to identify only specific regions of interests (ROI), 3 channels for each hemisphere have been selected, specifically: ch 1 (Fz-F1) and ch 2 (Fz-F2) for left and right dmPFC; ch 5 (F3-FC3) and ch 8 (F4-FC4) for left and right DLPFC; ch 15 (F7-FT7) and ch 17 (F8-FT8) for left and right for lOFC.

### 2.4. Statistical Analysis

The statistical analyses performed for this study included two parts: stimuli validation and the evaluation of significant differences in their fNIRS hemodynamic responses among the experimental conditions. Descriptive statics were used to study ratings and behavioral responses during the validation study. Furthermore, in this part, mix-design ANOVAs were used to evaluate significant differences.

To compare the hemodynamic activity of the participants based on the different independent variables (including task condition, offer type, and gender), we performed two mixed-design ANOVAs to O2Hb and HHb values (as analyzed in Section 2.4) with 4 repeated factors (condition: E, M; offer: 2: D, U; region of interest: dmPFC, DLPFC, lOFC, and hemisphere: left, right) and 1 between factor (gender: M, F).

Shapiro–Wilk tests were computed to check the normality of variables. For all of the ANOVA tests, degrees of freedom were corrected by Greenhouse-Geisser epsilon, where appropriate. All the analyses were performed using the SPSS package (version 23.0, IBM, Armonk, NY, USA, 2014).

### 2.5. Stimuli and Stimuli Validation

Before beginning with the experimental data acquisition, stimuli were tested with a preliminary validation phase. Sentences were evaluated by a group of 18 judges (9 males and 9 females) of a comparable age (range = 24–35; M_f_ = 29.7; SD_f_ = 7.65. M_m_ = 26.3; SD_m_ = 2.18) and education (range = 13–18; M_f_ = 15.9; SD_f_ = 2.32. M_m_ = 16.6; SD_m_ = 2.6) with respect to the experimental group.

A first analysis was meant to check the behavioral effects related to some variables of interest, thus excluding possible confounding effects. The judges were required to express their level of agreement to 6 questions by using a 5-point Likert scale ranging from 1 (I do not agree at all) to 5 (I completely agree). Questions were aimed at assessing sentences’ comprehensibility, the difficulty in making a decision, sentences’ moral content, the easiness to identify with the situation about both the own subject and other significant people, and the easiness to the presence of conflict in making a decision. Every experimental condition (from 1 to 6) was presented on a single slide by following the same structure described in the procedure section (see Section 2.2). The scenario was typed in black Calibri 22 font. Thus, 6 mixed-design ANOVAs have been applied to the dependent variables with condition (2: E, M) and offer (3: N, D, U) as repeated factors and gender (2: M, F) as the between factor. The analyses revealed significant effects for the presence of **moral content**. In particular, ANOVA revealed a significant effect for Condition (F1,17 = 25.15; *p* < 0.0001; η^2^p = 0.6) with the judges expressing higher ratings for the presence of a moral content within the M (M = 4.26, SD = 0.21) than the E (M = 3.22, SD = 0.25) conditions. 

Moreover, the ANOVA about the presence of conflict revealed a significant effect for Offer (F2,34 = 6.22; *p* < 0.01; η^2^p = 0.27). Paired multiple comparisons revealed that the N offer was associated with the lowest level of agreement about the presence of conflict (M = 2.53; SD = 0.24) about the D offer (*p* < 0.05; M = 3.22; SD = 0.21). No significant differences emerged with the D offers, or between D and U offers. No other significant effects emerged from ANOVA concerning sentences’ comprehensibility, the difficulty to make a decision, the easiness of identifying with the situation concerning either the own subject or other significant people.

A second analysis was aimed at comparing judges’ tendency to accept or refuse the offers across the different experimental conditions. The “accept” answers were transformed into percentages within each experimental condition and submitted to another mixed-design ANOVA as dependent variable, with Condition (2: E, M) and Offer (3: N, D, U) as a repeated factor, and gender (2: M, F) as a between factor. Results showed a significant effect for Condition (F1,16 = 17.96; *p* < 0.005; η^2^p = 0.53). Judges were more inclined to accept offers within the M condition (M = 87.41%; SD = 5.33) than the E (*p* < 0.005; M = 59.63%; SD = 6.57) condition. Also, the analysis revealed a significant effect for Offer (F2,32 = 22.01; *p* < 0.0001; η^2^p = 0.58). Paired multiple comparisons revealed that judges were less inclined to accept D offers (M = 53.89%; SD = 6.04) than N (*p* < 0.0001; M = 88.89%; SD = 5.39) and U ( *p*< 0.005; M = 77.78%; SD = 6.21) offers. No significant differences emerged between N and U offers.

Moreover, the analysis revealed a significant Gender * Offer effect (F2,32 = 3.53; *p* < 0.05; η^2^ = 0.18). Paired multiple comparisons revealed that males were more inclined (*p* < 0.05) to accept U offers (M = 94.44%; SD = 8.78) than females (M = 61.11%; SD = 8.78).

**Figure 3:**
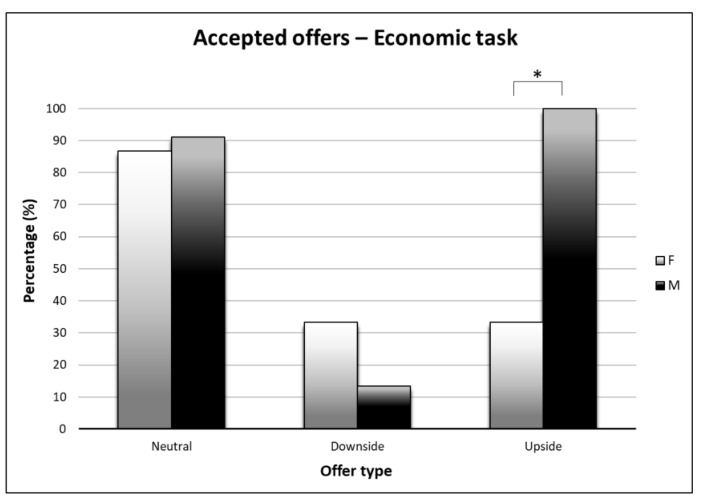
Percentage of accepted offers by males and females for condition and offer type. Statistically significant comparisons are marked with an asterisk.

Finally, the analysis revealed a significant Gender * Condition * Offer effect (F2,32 = 9.83; *p* < 0.0001; η^2^ = 0.38). Paired multiple comparisons revealed that such effect was mainly present in the E condition, where males judges were more inclined (*p* < 0.005) to accept U offers (M = 100%; SD = 11.79) than females judges (M = 33.33%; SD = 11.79) (see Figure 3).

## 3. Results

For what concerns O2Hb values, the ANOVA revealed a significant effect for Offer (F1,31 = 8.95; *p* = 0.005; η^2^p = 0.23) with higher O2Hb levels for D (M = 0.05; SE = 0.03) than U offers (M = −0.02; SE = 0.02). 

**Figure 4:**
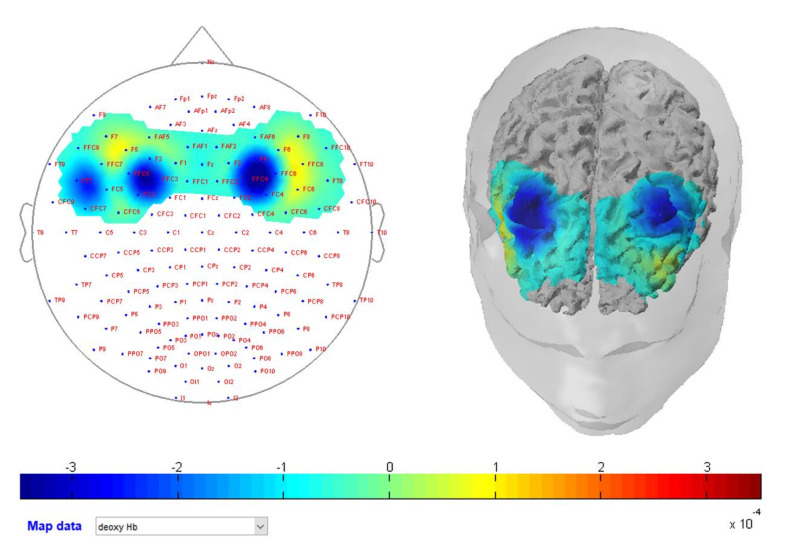
Hemodynamic map of one exemplificative woman participant. The figure shows a decrease in HHb level over the DLPFC during the economic condition. The frame was recorded in response to upside offers.

For what concerns HHb values, the ANOVA revealed a significant interaction effect for Condition * ROI * Gender (F2,60 = 4; *p* = 0.02; η^2^p = 0.12). Paired multiple comparisons revealed that the HHb level of female participants within the DLPFC was decreased during the economic condition (M = −0.1; SE = 0.04) concerning the moral one (*p* = 0.02; M = 0.01; SE = 0.03) (see Figure 4).

**Figure 5:**
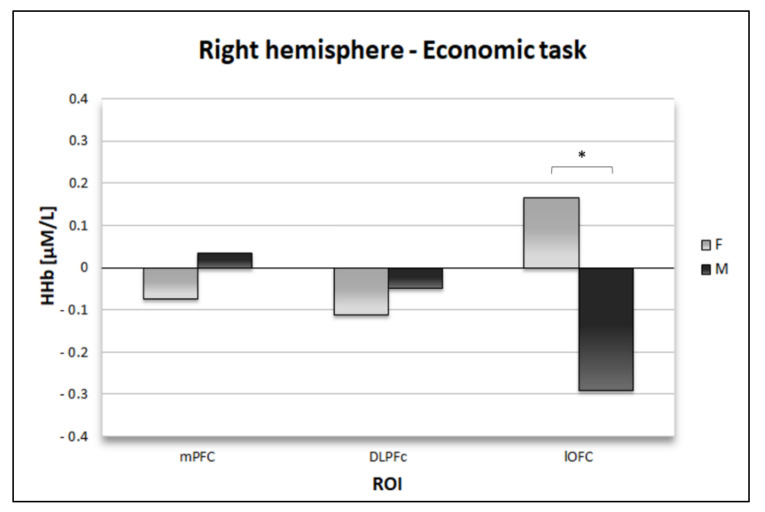
Histogram representing differences in the right-hemisphere brain activity between males and females during the economic task. Statistically significant comparisons are marked with an asterisk.

Also, the analysis revealed a significant Condition * Offer * Side effect (F1,30 = 6.91; *p* = 0.013; η^2^p = 0.19). Paired multiple comparisons revealed that the deoxy activity elicited by advantageous moral offers (U) was lower over the right (M = −0.17; SE = 0.04) than the left hemisphere (*p* = 0.002; M = 0.12; SE = 0.05). Moreover, a significant Condition * ROI * Side * Gender interaction effect emerged (F2,60 = 4.42; *p* = 0.02; η^2^p = 0.13). Paired multiple comparisons revealed that the deoxy activity elicited during the economic condition over the right lOFC was lower for male participants (M = −0.3; SE = 0.18) than females (*p* < 0.05; M = −0.08; SE = 0.15) (see Figure 5). 

Also, the deoxy activity of males in this area was lower during the economic condition (M = −0.3; SE = 0.18) with respect to the moral one (*p* = 0.006; M = 0.19; SE = 0.13). Finally, during the moral condition, male participants showed lower deoxy levels over right (M = −0.12; SE = 0.06) than left DLPFC (*p* = 0.005; M = 0.1; SE = 0.05).

## 4. Discussion

The present study aimed at exploring the brain responses of participants while playing 2 different versions of a modified UG, presenting an economic or a moral frame, as well as advantageous (U) and disadvantageous (D) offers. The main hypothesis was that the framing of the task could be effective in modifying conflict processing and that specific prefrontal activations could mediate these differences. Moreover, we were interested in investigating the role of gender in modulating such effects.

We first evaluated each decisional frame used to assess content validity, in particular for the moral content of the scenarios and conflict processing. Thus, we had the confirmation that: (1) the moral frame was considered having moral content; (2) that the conflicts depicted in each frame were even regarded as conflictual (i.e., involving a complicated decision-making process); (3) that the conflicts presented within the moral frame were considered having a higher moral content than the conflict presented within the economic frame.

About the behavioral responses to the offers, results revealed that in all frames, 50–50 offers were the easiest to accept, followed by advantageous (U) and disadvantageous (D) ones. Males were more inclined to get advantageous offers, while females were more inclined to accept disadvantageous offers.

Moving to neurophysiological data, we wanted to test if the conflictual content of offers was associated with a specific activation pattern of the PFCs and how the decision frame modulated it. We found some interesting outcomes: first (I), we found that disadvantageous offers (D) were associated with an increased general cortical activation. Secondly (II), we found that advantageous moral choices at the expense of others (U) were related to the activation of the right prefrontal cortex. Finally (III), we found the recruitment of specialized neural networks for males and females in the different frameworks.

First, the analyses revealed an increased cortical activity when processing disadvantageous offers (D). We hypothesized that this kind of offer could be associated with increased cognitive conflict and, subsequently, a higher cognitive load. Indeed, the participants knew that, if they refused the offer, they would lose everything and gain zero euros. On the other side, they had to consider taking a minimal remuneration when sharing money both with a colleague or with a charitable association. Our hypothesis is coherent with previous fNIRS research, which already demonstrated the efficacy of such techniques in identifying prefrontal modulation in response to mental workload (see, for example Reference [52]).

Secondly, we found that considering advantageous moral offers (U) led to the recruitment of the right prefrontal sites. According to the frontal asymmetry hypothesis, which describes the left hemisphere as devoted to positive emotion processing and approach attitudes, and the right one to negative emotions and avoidant behaviors, we could hypothesize that within a moral condition, the idea of making more money when sharing with a charity association could be associated with some bad feelings such as regret and inequity aversion. Here, we might infer that advantageous offers, especially in the moral frame, were accompanied by emotional conflict. Differently from our hypotheses, the effect was present in both males and females, but only in the moral frame. We could assume that when the content of the unfairness is related to moral issues, both males and females experience a high conflict situation and perceive the presence of injustice negatively. We can interpret this effect as due to a social/emotional conflict that is more related to inequity aversion rather than a cognitive issue.

Thirdly, and coherently with our main hypothesis, we found significant effects related to gender differences. However, results only partially supported our predictions, and they have not a straightforward interpretation. In detail, we found that the deoxy-Hb level of females showed a higher decrease during the economic condition than the moral one over the dorsolateral regions. Since a reduction of deoxy-Hb is associated with an increase of the same areas as measured by the BOLD signal [53], we may argue that for females, the DLPFC was more consistently recruited during economic frames than the moral ones. This could be coherent with the view that DLPFC is mainly involved in strategic decisions and, anyway, in all tasks that require the cognitive integration of different data. Indeed, as previously discussed, the DLPFC is thought to facilitate strategic decisions over riskier choices [24]. For example, previous TMS studies demonstrated that when the DLPFC is inhibited, risk-taking behaviors increase [25]. Thus, future studies should address the hypothesis that females perceive economic frames as potentially risky and subsequently engage in strategies to rule them.

On the other hand, during the economic frame, males engaged more in the orbitofrontal areas (lOFC). Previous studies reported lOFC to be activated by choices involving possible losses [30]. Even if the orbitofrontal area has also been associated with processing large gains [26], dissociation has been found between medial and lateral portions of the OFC. In detail, the medial areas proved to be correlated with monetary gain and the lateral regions with the monetary loss [29]. Thus, we could propose that males evaluated economic offers in light of gain/loss considerations to avoid future regret [54]. Alternatively, we could hypothesize that they assessed the economic offers as more emotionally conflictual [29] than females did, with a modulation of the prefrontal network. Indeed, the lOFC is also thought to lo be linked to the inhibitory control of emotion [55].

On the other hand, during the economic frame, males recruited the lOFC. As we described above, this area is involved both in reward-related mechanisms and emotional aspects of decision-making [55]. Consequently, it is possible that the lOFC helped males avoiding the interference of negative emotions so to obtain better monetary results.

Furthermore, during the moral frame, males engaged more in the DLPFC, which is the same circuit that females recruited for economic choices, but in this case, the activity was mainly over the right hemisphere. The balance between right and left DLPFC is thought to be critical for adaptive decision-making (for review, see References [24,56,57]). Repetitive transcranial magnetic stimulation studies have shown that the disruption of the right DLPFC increases risk-taking behavior [25]. In contrast, a left anodal/right cathodal tDCS over the DLPFC decreases subjects’ risk-taking [24]. These results indicated that the increase in relative right frontal activity caused participants to be more attracted by safer choices. This way, manipulating the increase in the right frontal activity mitigates the power of larger, less likely rewards in driving participants’ behavior. In few words, if females recruited more this strategic circuit for economic decisions, males relied on it for moral frames, probably perceived as linked to potential social negative rewards, and thus most in need of cognitive control and verification. Therefore, the activation of the right DLPFC in males could mean that they need focused processing of the moral frame in search of a safer decision. Instead, the reason why females activate the DLPFC bilaterally during economic frames than males may be found in their need to integrate data, evaluating negative and positive aspects, and to arrive at a strategic decision, a process linked to frontal symmetry.

## 5. Conclusions

Our results highlighted that disadvantageous conflict was associated with increased prefrontal activation. Disadvantageous offers could be associated more with a cognitive conflict, which involves cost/benefits analysis. Thus, they are associated with a higher cognitive load. On the other hand, advantageous offers are related to the recruitment of a right-sided network related to negative emotions and avoidant behaviors. Indeed, the effect was present only for moral offers, which involved taking more money for themselves and giving less to a charitable association. We can, thus, interpret this as a social/emotional conflict that is related to inequity aversion.

Moreover, in agreement with our hypotheses, we found gender differences based on the scenario. Specifically, the DLPFC was recruited by females during the economic task, and by males during the moral frame. Since this area has been associated with risk perception and the adoption of safe behaviors, we can suppose that females and males displayed a different perception of these different frames. This is in line with previous research indicating a different decision-making style according to gender, with females being more prosocial, and males more rational and pragmatic [47].

The present study presents some possible limitations. The first one involves the absence of behavioral data due to the lack of variability in responses. In fact, participants have systematically either accepted or rejected the answers based on the type of offer. This behavior pattern is related to the low ecological quality of the task. Previous studies already highlighted how computerized negotiations are very different from real face-to-face UG. They suffer from the presence of cognitive biases related to the absence of a real opponent. This fact could also be supported by the block design paradigm, which could have made the task repetitive. Future research could, thus, look for more ecological and real-life solutions.

Furthermore, we did not control participants’ general and moral intelligence, which could modulate decision-making processes as well as the involved neural circuits. Finally, since fNIRS can record only the hemodynamic activity coming from the brain surface, other techniques could be used to investigate better the role of cognitive and emotional mechanisms linked to subcortical circuits.

However, despite these limitations, we believe that our study proved to be useful to compare two different conflict framings systematically and to identify dedicated brain networks based on various cognitive and emotional strategies, and specifically for males and females. Future research could apply the same protocol in another real-life context, such as medical decision-making, marketing, business, or management dynamics.

## Figures and Tables

**Table 1:** Conditions (Economic, Moral) and offers (Neutral, Downside, Upside) presented during the experiment.

	Neutral	Downside	Upside
Economic	You have worked as much as your colleague for a project paid 1000€. The offer is about 50% both.	You have worked as much as your colleague for a project paid 1000€. The offer is about 20% for you and 80% for your colleague.	You have worked as much as your colleague for a project paid 1000€. The offer is about 80% for you and 20% for your colleague.
Moral	A 1000€ money surplus from your company is equally (50%) divided between you and a charity association that supported your colleague’s son/daughter.	Your 1000€ annual bonus is divided between you (20%) and the charity association (80%) that supported your colleague’s son/daughter.	Your 1000€ annual bonus is divided between you (80%) and the charity association (20%) that supported your colleague’s son/daughter.
